# Audit of Rapid Tranquillisation Prescribing and Monitoring Practices at Rohallion Medium and Low Secure Forensic Psychiatry Unit, Murray Royal Hospital, NHS Tayside

**DOI:** 10.1192/bjo.2025.10693

**Published:** 2025-06-20

**Authors:** Aditya Unni, Oana Maior, Jonathan Bate

**Affiliations:** Rohallion Unit, Murray Royal Hospital, NHS Tayside, Perth, United Kingdom

## Abstract

**Aims:** To assess whether current prescribing and monitoring practices for oral 'as required’ medications and Rapid Tranquillisation align with local and national guidelines.

To identify areas of non-compliance and enhance awareness of best practice guidance.

**Methods:** The audit included all patients at Rohallion Clinic, Perth, who had 'as required’ medications prescribed for sedation, anxiety, agitation, or behavioural disturbance at the time of data collection. Female, child, and adolescent patients were not included, as these populations are not present in Rohallion Clinic.

Data collection: Data were collected using an audit proforma during the period between 05/03/2024 and 04/09/2024 of 47 inpatients. Patients’ online drug charts and EMIS (electronic notes) were reviewed using MS Excel.

Standard:

1. 100% of patients should have a documented plan for oral and intramuscular 'as required’ medication in the notes, including if more than 1 medicine is required.

2. 100% of patients should be offered oral medication, if practicable, before administration of intramuscular medication.

3. 100% of patients should have side-effects monitored within 1 hour of rapid tranquillisation. If not possible, this should be documented on the observations chart and in the notes.

Criteria:

1. Multidisciplinary teams should develop and document an individualised pharmacological strategy for using calm, relax, tranquillise or sedate patients who are at risk of violence and aggression.

2. Oral medicines should be offered first, if practicable, before intramuscular medication.

3. After rapid tranquillisation, monitor side-effects, observations, level of hydration and level of consciousness at least every hour until there are no further concerns regarding physical health.

**Results:** NHS Tayside’s guideline on the pharmacological management of acute behavioural disturbance was updated in Oct 2024.

Total 81% (38) patients had 'as required’ medicines prescribed on the drug chart.

Lorazepam was prescribed most frequently. This is in line with NHS Tayside guidelines which consider lorazepam the first strategy for management of acute behavioral disturbance.

63% (24)of patients (who were on 'as required” medications) had a documented plan.

Standard 1 is not met.

The reasons for administering intramuscular as-required medications, along with documentation of side effect monitoring and observations, including any reasons for omissions, were recorded in electronic notes 25.42% of the time.

Therefore, standard 2 and standard 3 are not met.

**Conclusion:** One area identified as compliant with current NHS Tayside guidance is the frequency of medication administration, with most medicines prescribed every 4 hours.

Our data shows that lorazepam, promethazine, and haloperidol are the most commonly used medications, with fewer newer medications being prescribed.

Standards 1, 2, and 3 are not met.

Action plan:

Collaborate with the clinical team and pharmacist to improve the accuracy and completeness of documentation related to medication administration, including consent, administration records, and observed effects.

Add additional headers to online assessment templates to support more comprehensive documentation.

Re-audit once the action plan is implemented to assess any changes.

